# Interactive effects of planting patterns combined with integrated nutrient management on maize production, water-nitrogen productivity and soil organic carbon fractions

**DOI:** 10.1186/s12870-025-06456-3

**Published:** 2025-04-26

**Authors:** Wenting Jiang, Zhongyang Wang, Baodong Chen, Jifu Ma, Nana Bao, Guoliang Chen, Xiukang Wang, Yuting Cheng

**Affiliations:** 1 State Key Laboratory of Urban and Regional Ecology, Research Center for Eco-Environmental Sciences, Chinese Academy of Sciences, Beijing, 100085 China; 2 Research and Development Centre of Ecological and Sustainable application of Microbial Industry of the Loess Plateau in Shaanxi Province, Yan’an University, Yan’an, Shaanxi China

**Keywords:** Temperature, Soil water storage, Water productivity, Nitrogen, Entropy TOPSIS model

## Abstract

**Background:**

Ridge and furrow rain-harvesting planting system and integrated nutrient management are crucial strategies for enhancing soil moisture and fertility in drylands. However, the synergistic impact of these approaches on soil hydrothermal environment, maize productivity, water-nitrogen productivity and soil carbon fractions has not been fully understood. A two-year field experiment were conducted in 2020 and 2021 was undertaken in Loess Plateau of China. Six treatments: (i-iii) Conventional flat planting with no nutrient management (CP), with nitrogen fertilizer (CPN), with nitrogen fertilizer and maize straw (CPSN); (iv-vi) ridge and furrow rain-harvesting planting with no nutrient management (RF), with nitrogen fertilizer (RFN), with nitrogen fertilizer and maize straw (RFSN).

**Results:**

The results showed that the RF, CPN, RFN, CPSN and RFSN significantly improved soil temperature, which showed gradually increased after the seedling stage and slowly decreased at silking to maturity stage in two maize growing period. The dynamics of soil water storage (SWS) varied significantly throughout the six growing periods. The increasing of grain yield and above-ground biomass were highly influenced by ridge and furrow rain-harvesting planting system and interaction with nitrogen fertilizer and crop straw management. The grain and straw N content, plant N uptake, water productivity also similar with the rank of grain yield and above-ground followed by RFSN>CPSN>FPN>CPN>FP>CP. Furthermore, the RFSN treatment significantly increased the N uptake efficiency (NUPE), agronomy efficiency of N (AEN), and partial factor productivity of N fertilizer (PFPN) compared to RFN, with a mean increase of 29.2%, 44.5% and 7.8% in both growing years. Meanwhile, the RFSN treatment increased DOC by 51.1% (53.2%), EOC by 25.4 % (26.1%), MBC by 96.3% (80.8%), MBN by 52.6% (86.7%) in 2020 (2021), respectively.

**Conclusions:**

As a result, gray relation analysis and entropy TOPSIS model evaluated that application of maize straw 1.5 t ha^-1^ and chemical nitrogen fertilizer of 180 kg ha^-1^ in ridge and furrow rainwater harvesting system (RFSN) serve as a effective method of soil management to enhance water and nitrogen utilization, potentially boosting yields and promoting resource efficiency in the arid regions of the Loess Plateau.

## Background

Agriculture faced unprecedented challenges as global climate change intensifies and water resources continue to diminish [1]. Arid lands occupied nearly half of the earth's land, which makeing dryland agriculture increasingly crucial [2]. Dryland farming must overcome natural environmental challenges and address the growing pressure of food production to ensure stable global food supply. Low water availability and frequent drought stress occurs resulted in turn gravely threatens the food security of these areas owing to the high variability of rainfall in arid regions [3]. Therefore, improved water supply is crucial for stabilizing and increasing crop productivity.

Water scarcity is a critical global issue that limited agricultural productivity and financial growth in dry and semi-arid areas. Rain-fed agriculture is a important contributor to the cultivated land in China, accounting for 70% of the total land area [4]. The region experiences low and irregular seasonal precipitation, making crop water shortages extremely common. Moreover, the timing of precipitation not align with the water requirements of the growing crops, and water shortages and poor soil conditions severely restrict agricultural productivity and sustainability in the semi-arid regions of the Loess Plateau. Maize as one of the largest-planted and most economically valuable cereal crops in China, accounts for 41% of the total world grain production [5]. Despite the continuous introduction of higher yields and more adaptable maize varieties through breeding technologies, maize yield has still not fundamentally improved due to the influence of various factors such as water scarcity and soil infertility. Therefore, effective rainwater conservation measures and the improvement of water resource utilization planting patterns are crucial for local maize production and security.

The ridge and furrow rain harvesting (RFRH) system has great advantages in promoting crop productivity in dryland, and has emerged as one of the main water-saving irrigation planting patterns in arid regions of China [6]. The RHRF was altered the topography of the land surface, and augmented the area that receiving solar irradiation and curtailing the water exchange between the soil and atmosphere through the utilization of thin-film covering. This innovative approach not only addresses water scarcity but also creates a more favorable microenvironment for crop growth. Previous researches shown that the RFRH could collected surface runoff generated by rainfall and reduced ineffective evaporation, and improve soil water status in crop growing areas [7]. RHRF not only suppresses soil moisture evaporation but also directs rainfall into furrowed catchments via runoff to facilitated for crop growth in regions with substantial evapotranspiration, [8]. This system also had a runoff effect on the rainfall of 1mm, and increasing soil water storage in soil layer at all stages of crop growth. The main characteristics of RHRF are its soil warming and water replenishing effects. RHRF effectively reduced the latent heat flux and elevated the temperature at the soil surface. Previous studies have also indicated that the RHRF not only ameliorates soil thermal and moisture conditions but also enhances crop growth and development. The model provides excellent moisture support and create favorable conditions for crop photosynthesis, which significantly increased the photosynthetic rate more than 10-67% compared with the flat planting system [9]. RFRH is widely used by various types of plants, and therefore a critical planting system for ensuring food security in dryland growing area in the world.

Integrated nutrient management is vital in fortifying and refining the physicochemical characteristics of dryland soil, which is not only related to crop growth and soil fertility sustenance and augmentation, but also a pivotal measure for realizing agricultural circular economy and facilitated agricultural sustainable development. The application of crop straw and fertilizer plays an important role in integrated nutrient management. Crop straw, being a vastly accessible and directly exploitable renewable source of organic cellulose, and replete with carbon, nitrogen, phosphorus, potassium, and an array of trace elements [10]. Especially in crop straw is returned to the field, it can promote soil organic carbon sequestration, and then effectively improve soil chemical properties. Xu et al. [6] found that full or half corn straw could significantly increase soil organic carbon storage by 52.5% and 31.7%, respectively. Further research by wang et al. [11] indicated that straw restitution can improve the soil microhabitat by encouraging the development of soil aggregates, and augment the quantity of organic matter sequestered within these aggregates and microaggregates, and diminished the microbial decomposition of organic carbon and substantially increasing the carbon's stabilization in the soil. The strategic addition of straw has been shown to amplify soil fertility by elevating the levels of soil active organic carbon and enzymatic activity. Straw addition can improve soil fertility by increasing soil active organic carbon enzyme activity. Moreover, fertilization as plays a crucial role in enhancing food production and ensuring food security in dryland areas. In semi-arid regions, the application of 90 kg N ha of nitrogen fertilizer alone can result in a significant 109% increase in maize yields [12]. This increase is attributed to the low total nitrogen content of soils in the region. However, current grain yields heavily rely on excessive fertilizer application, raising environmental concerns. Effective nutrient management needs to consider soil type, crop demand, regional climate, and environmental protection,with its succes large dependent on soil moisture.

In recent years, the synergistic effect of integrating nutrient management and water availability has attracted significant attention. However, little is known about the combined impacts of the ridge and furrow rain harvesting (RFRH) system and integr ated nutrient management on resource use efficiency and yield potential. Field trials have not fully explored the inteplay between the RFRH system, crop straw, and nitrogen fertilizer. Further investigation is needed to assess whether the RFRH system could serve as an effective control measure and how it could be integrated with nutrient management to optimize agricultural production and enhance crop water-nitrogen use efficiency.

Previous studies have focused on the individual effects of RFRH [13] or RFRH with fertilizer [14] or biochar [15] on soil nutrition, economic benefits of ecosystems, and crop productivity. It is first time to research whether RHRF combining these factors can improve soil fertility and yield benefits in dryland areas. Therefore, we hypothesize that combining straw and nitrogen fertilizer under RFRH system could enhance maize yield and water-nitrogen use efficiencies simultaneously. The integrated approach aims to maximize resource utilization while promoting sustainability. The purpose of this study was to explore (i) to compare the interaction effects of different planting patterns coupled with integrated nutrient management on soil temperature, soil moisture dynamics, yield, biomass, harvest index, grain and straw N content, plant N uptake, water productivity (WP), nitrogen uptake use efficiency (NUPE), agronomy efficiency (AE) and partial factor productivity of nitrogen (PFPN) and soil C/N fractions; (ii) analyze the relationship between soil hydrothermal characteristics, yield components, water and nitrogen productivity and soil organic carbon fractions; and (ii) determine which as optimized cultivation practice for improving soil environment and maize production on the Plateau. We hypothesized that the ridge and furrow rain harvesting planting combined with the utilization of integrated nutrient management has the potential to beneficial for soil hydrothermal to enhance the crop productivity, resource utilization efficiency. A comprehensive comprehension of the impact of soil environment on crop yield can serve as a theoretical foundation for maintaining soil fertility and attaining sustainable dryland agricultural development.

## Methods

### Description of the experimental site

**Fig. 1:**
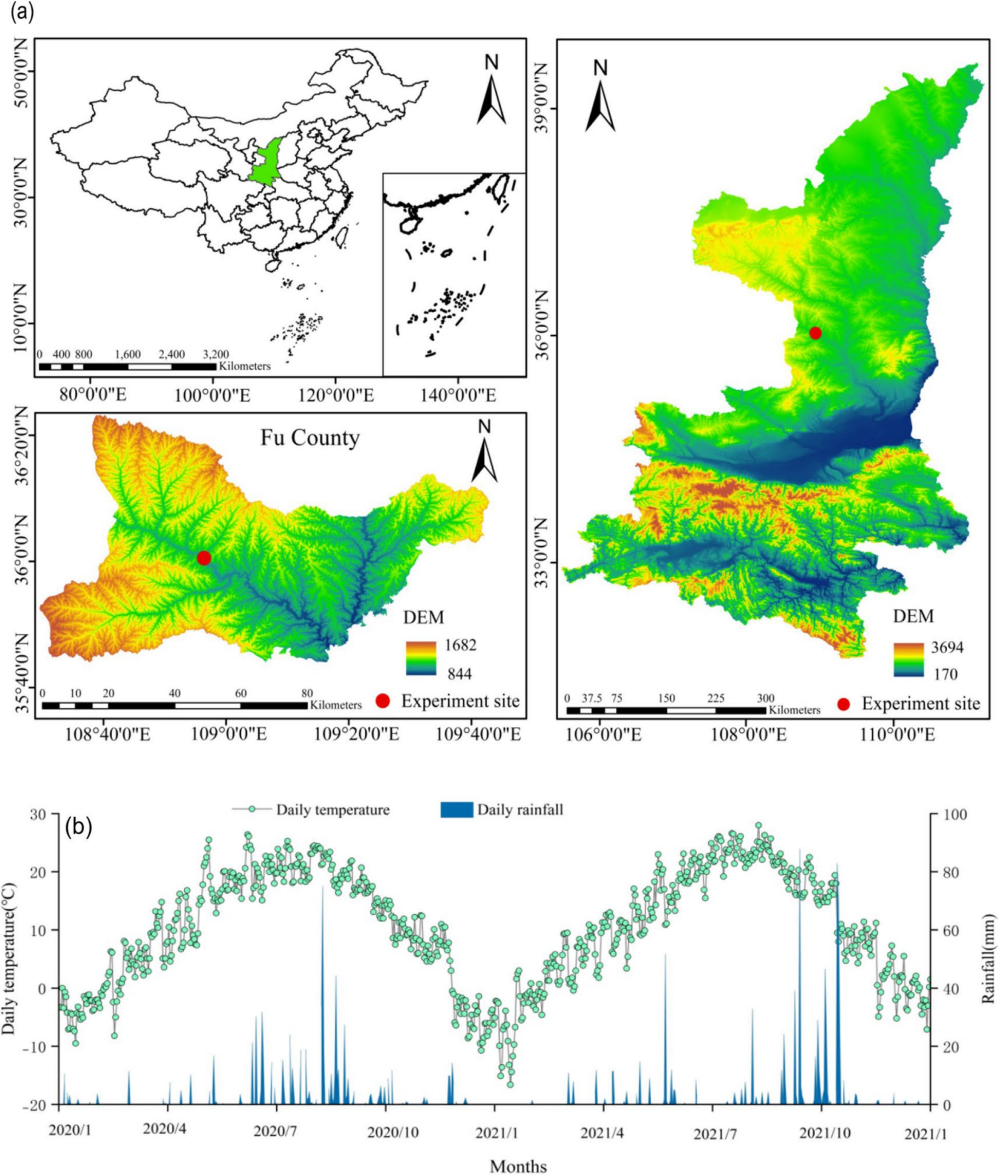
Location of experimental site in Fu xian county of Shaanxi province in China (**a**) and the Variability patterns of monthly mean temperature and rainfall at the experimental site during the period of 2020 - 2021 (**b**)

The experiment was conducted in 2020-2021 at the Farming experimental site (36°0'31" N, 108°56'25" E, 1057m above sea level) in Fuxian County, Yan 'an city, Shaanxi Province (Fig. 1a). The region is a typical warm temperate continental monsoon climate, with an average annual temperature of 8.9℃, an average annual evaporation of 1050 mm, and a 40-year average precipitation (1980-2021) of 580 mm (Fig. 1b). The annual rainfall generally lasts from April to early October. During the experiment period, precipitation fluctuated greatly. The total precipitation during the growing period of maize ranged from 472.5 mm to 506.2 mm, and the two-year average was 489.3 mm. The soil texture of the experiment site was yellow spongy soil, and the physicochemical properties of the surface soil (0-20 cm) before sowing were as follows: organic matter of 12.3 g kg^-1^, total nitrogen of 0.8 g kg^-1^, available phosphorus 6.51 mg kg^-1^, available potassium of 98.25 g kg^-1^, alkali-hydrolyzed nitrogen of 114 mg kg^-1^, pH 7.6.

### Experimental design and field management

**Fig. 2:**
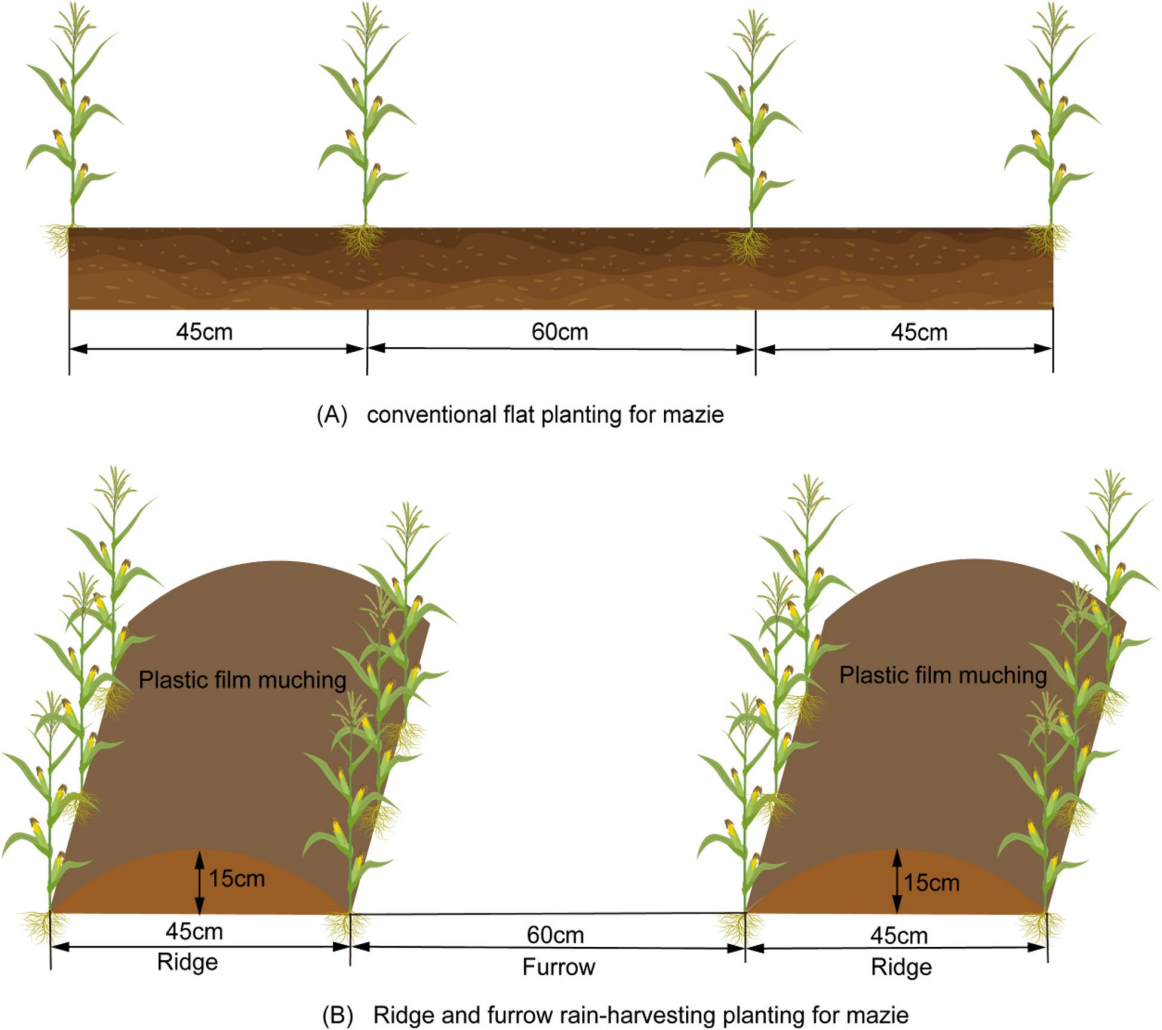
Field layouts of the conventional flat planting (**A**) and ridge-furrow rainwater harvesting system (**B**)

The field trial was conducted from April 2021 to October 2022 in a randomized complete block design with three replicates. The two planting patterns include (i) conventional flat planting (CP, Fig. 2A); (ii) ridge and furrow rain-harvesting planting (RF, Fig. 2B). Both system with three nutrient management treatments (CK, control; N, only application nitrogen fertilizer rates of 180 kg ha^-1^; NS, application of maize straw 1.5 t ha^-1^ and chemical nitrogen fertilizer of 180 kg ha^-1^). There were six treatments: conventional flat planting without nutrinet management (CP), ridge and furrow rain-harvesting planting without nutrinet management (RF), conventional flat planting with only application nitrogen fertilizer rates of 180 kg ha^-1^ (CPN), ridge and furrow rain-harvesting planting with only application nitrogen fertilizer rates of 180 kg ha^-1^ (RFN), conventional flat planting withapplication of maize straw 1.5 t ha^-1^ and chemical nitrogen fertilizer of 180 kg ha^-1^ (CPSN), conventional flat planting with application of maize straw 1.5 t ha^-1^ and chemical nitrogen fertilizer of 180 kg ha^-1^ (RFSN). The rates of chemical nitrogen fertilizer were urea (46%N ), and the rates of 150 kg ha^-1^ N. The rates of P and K fertilization were 90 kg ha^-1^ P and 120 kg ha^-1^ K, which were calcium superphosphate (12% P) and potassium chloride (60% K), respectively. The maize straw was chopped (1-2 cm) were uniformly plowed into the soil layer (0-20 cm), and the soil was deposited by a rotary machine in October 2019. Before maize sowing period, the soil of 30cm was plowed, chemical fertilizer in CF, MS and MC treatments were applied into the soil as basal fertilizer, and the treatments in the ridge and furrow rain harvesting planting system were covered with plastic film. The RFRH system used ridge and furrow widths of 45 cm, which ridge height was 15 cm and the ridges were covered with ordinary transparent and impermeable plastic film (film width 80 cm and thickness 0.02 mm). The conventional flat planting system (Fig. 2B) is not covered with film, which belongs to normal cultivation. Each plots had an area of 61.2 m^2^ (17 m× 3.6 m). The spring maize variety ‘Liaodan 352′ was purchase in market and sown on on April 28, 2020 and April 29, 2021 and harvested on October 2, 2020 and October 5, 2021. Urea (46% N), calcium superphosphate (16% P), and potassium sulfate (50% K) were using a hole-sowing machine with a seeding depth of 5-6cm and harvested in late September or October each year, and remove the mulch after harvested. In the semiarid rain-fed region, no irrigation was provided over the course of two experimental years. To prevent maize yield loss, weeds, pests, and diseases are controlled using conventional local field management practices (insecticides and herbicides).

### Measurement and calculations

#### Determination of biomass and maize yield

Ten plants were chosen randomly from each plot for biomass measurement during the seeding, jointing, tasseling, silking and pollination, filling, and harvesting stages. Initially, the plant components underwent dehydration at 105 °C for half an hour to suspend natural processes. Subsequently, they were subjected to further drying at 75°C until a stable weight was attained (ensuring the grain yield was normalized to 14% moisture content) and the weight of one thousand grains was documented.

#### Gravimetric soil water content, soil water storage and soil temperature

Soil samples were collected from the 0-80cm soil layers in each plot at various growth stages: seeding, jointing, tasseling, silking, pollination, and filling, respectively. Samples were collected during the seeding stage and at 20-day intervals throughout the growing season. In cases of rainfall on the scheduled sampling day, the sampling was postponed by 3-5 days. Soil samples were taken in 10cm layers using a soil drill with a 4cm diameter. For the RFRH treatments, samples were taken from the middle of the ridges and furrows, as well as the sides of the furrows. In the flat planting treatments, soil cores were collected adjacent to the rows and between the rows. Samples collected were transported to the laboratory using ice bags at the base and weighed within 2 hours of collection to measure the wet weight. The soil water content (gravimetric, SWC) was then determined using the oven-drying method. Soil water storage (SWS, mm) were calculated by following formula [16]:

1 $$\text{SWS }(\text{mm})=\text{ SWC}\times \text{BDi }\times \text{ hi}\times 10$$

Where C was the soil water content (gravimetric, SWC, %) , BD_i_ was soil bulk density (g cm^-3^) and h_i_ refers to as soil depth (mm), and i=1, 2, 3, 4, 5, 6 represented the soil layer of 0-10cm, 10-20cm, 20-30cm, 30-40cm, 40-50cm, and 50-60cm.

Soil temperature (℃) was meticulously monitored between the roots of two maize plants within the same row in each plot by utilizing a mercury geothermometer. Throughout the period spanning from 2021 to 2022, soil temperature measurements were diligently conducted at seeding, jointing, tasseling, silking, filling, and maturity stages at 5-day intervals. The soil temperature at a depth of 10cm was observed at three-hour intervals from 9:00 am to 17:00 pm on the day. The resultant data was used to calculate daily average soil temperatures, comprising the mean values recorded at 9:00, 12:00, 15:00, and 18:00.

### Plant N concentration and plant N uptake

Above-ground plants are separated into root and crop residues, which include stems and leaves. The grain and straw samples were passed through a 0.5 mm sieve for grinding. The concentrations of nitrogen (N) are subsequently quantified during corn harvesting. The dried samples are individually processed through grinding and digestion using H_2_SO_4_-H_2_O_2_. The concentrations of nitrogenare determined using the Kjeldahl method. The total N uptake was calculated by dry weight multiplying the N concentration of each part.

### Evapotranspiration and water productivity

WP (kg ha^-1^ mm^-1^) were estimated as follows:

2 $$\text{WP }=\text{ GY}/\text{ET}$$

where CWP is the water productivity (kg ha^-1^ mm^-1^), GY is the grain yield (kg ha^-1^), and ET is the evapotranspiration (mm).

### N use efficiencies

N agronomic efficiency (AEN, kg kg^-1^) were calculated for each plot using the following equations:

3 $$\text{AEN}=\text{YN}-\text{Y}0/\text{F}$$

Where YN is the grain yield in N fertilization treatments (kg ha^-1^), Y0 is the grain yield in without N fertilization (kg ha^-1^), F is the total amount of applied N (kg ha^-1^).

The Harvest index (HI, kg kg^-1^) was calculated as follow:

4 $$\text{HI}=\text{Grain yield}/\text{Biomass}$$

The uptake efficiencies for N (NUPE) were calculated using the following equations:

5 $$\text{NUPE}=\text{N uptake}/\text{ N application rates}$$

The partial factor productivity of N (PFPN) was calculated as follows:

6 $$\text{PFPN}=\text{Grain yield}/\text{ N application rates}$$

### Soil C and N fractions analysis

Soil samples were collected after maize harvest in 2020 and 2021. Ten sampling points were randomly selected from each plot and collected with a soil auger. The soil collected from each plot was homogeneously mixed and the fresh soil samples were passed through a 2 mm sieve and divided into two portions. One portion was stored in a refrigerator at 4°C for subsequent analysis of soil for DOC, EOC, MBC, and MBN content. The samples were air-dried for the assessment of soil physico-chemical properties.

The soil was evenly mixed with deionized water (1:5) and shaken for 1 hour. The mixture was then passed through a 0.045um filter membrane to determine dissolved organic carbon (DOC) using a TOC-LCPH soil total carbon analyzer. Easily oxidizable organic carbon (EOC) was quantified through oxidation using 333 mmol L^-1^ KMnO_4_ repercussion method. Soil microbial biomass carbon (MBC) and nitrogen (MBN) was measured by the chloroform fumigation-abstraction method. Briefly, soil sample was subjected to ethanol-free chloroform fumigation for 24 h, and abstracted with K_2_SO_4_, and the MBC content was measured using a TOC-LCPH soil total carbon analyzer.

### Model analysis

#### Gray relation analysis

Gray correlation analysis is a method that assesses the geometric proximity of behavioral factor sequences using sample data from each indicator. It aims to determine the closeness, magnitude, and order of correlation between each indicator factor. When the original data is not uniform, it needs to be dimensionless processing to make the data comparable. The maximum value (X0) of each index parameter is taken as the reference data, and the absolute value of the difference between the dimensionless value of each index parameter and the dimensionless value of the reference sequence is processed.

7 $${x{\prime}}_{i}\left(k\right)={x}_{i}(k)/ {x}_{0}$$

8 $${\Delta }_{i}\left(k\right)= \left|{x}_{0}\left(k\right)-{x}_{i}\left(k\right)\right|$$

Calculate the correlation coefficient by this formula:

9 $${\epsilon }_{i}=\frac{{min}_{i}{min}_{k}{\Delta }_{i}\left(k\right)+\rho \times {max}_{i}{max}_{k}{\Delta }_{i}\left(k\right)}{{\Delta }_{i}\left(k\right)+\rho \times {max}_{i}{max}_{k}{\Delta }_{i}\left(k\right)}$$

10 $${\varepsilon }_{i}= \frac{{\Delta }_{min}+{p\Delta }_{max}}{{\Delta }_{i}\left(k\right)+{p\Delta }_{max}}$$

Which $\varepsilon_1$ is the correlation coefficient of reference sequence and comparison sequence, ${min}_{i}{min}_{k}{\Delta }_{i}\left(k\right)$ is second-order minimum difference, ${max}_{i}{max}_{k}{\Delta }_{i}\left(k\right)$ is Second order maximum difference, the resolution coefficient $\rho$ is 0.5.

Calculation the correlation degree according to formula:

11 $${r}_{i}= \frac{1}{n} {\sum }_{k=1}^{n}{\varepsilon }_{i}\left(k\right)$$

### Entropy weighted TOPSIS established

The entropy-weighted TOPSIS model aims to minimize the influence of subjective factors on evaluation outcomes and enhance model accuracy. The TOPSIS method is a multi-objective decision-making analysis technique for a limited number of scenarios. Its main objective is to identify the optimal and worst scenarios based on the normalized original data matrix. The method then calculates the distance between each evaluation scenario and the optimal and worst scenarios, respectively, to obtain the relative proximity of the evaluation scenarios to the optimal scenarios. This serves as a basis for evaluation. The modelling process is as follows.

Assuming a multi-indicator decision-making problem with n evaluation indicators and m programmes, the programme set and indicator set are denoted as M = (M_1_, M_2_, ..., M_n_) and C = (C_1_, C_2_, ..., C_m_) respectively. X_ij_ (i = 1, 2, ..., n; j = 1, 2, ..., m) is the estimated value of indicator C for programme M.

12 $$X= \left[\begin{array}{cccc}{x}_{11}& {x}_{12}& \cdots & {x}_{1m}\\ {x}_{21}& {x}_{22}& \cdots & {x}_{2m}\\ \vdots & \vdots & \vdots & \vdots \\ {x}_{n1}& {x}_{n2}& \cdots & {x}_{m}\end{array}\right]$$

Using the normalisation of the judgement matrix, the following formula was used for the larger and smaller indicators, respectively.

13 $${x}_{ij}^{*}= \frac{{x}_{ij}}{\sqrt{{\sum }_{i=1}^{n}{x}_{ij}^{2}}}$$

14 $${x}_{ij}^{*}= \frac{1/{x}_{ij}}{\sqrt{{\sum }_{i=1}^{n}{\left(1/{x}_{ij}\right)}^{2}}}$$

Transform the matrix into a normalised matrix by dimensionless processing Zij (i=1, 2, ..., m; j=1, 2, ...,n), and further determine the positive ideal solution vector Z^+^= (z^+^_1_, z^+^_2_, ..., z^+^_n_) and the negative ideal solution vector Z^-^= (z^-^_1_, z^-^_2_, ..., z^-^_n_) of the matrix Z.

Calculate the euclidean distance (S_i_^+^ and S_i_^-^) of the evaluation programme from the positive and negative ideal solutions using the formula.

15 $$\begin{array}{cc}{S}_{i}^{+}= \surd \sum_{i=1}^{n}{\left({z}_{ij}-{z}_{ij}^{+}\right)}^{2}& (\text{i}=\text{1,2}, ...,\text{ n })\end{array}$$

16 $$\begin{array}{cc}{S}_{i}^{-}= \surd \sum_{i=1}^{n}{\left({z}_{ij}-{z}_{ij}^{-}\right)}^{2}& (\text{i}=\text{1,2}, ...,\text{ n })\end{array}$$

17 $$\begin{array}{cc}{C}_{i}= \frac{{S}_{i}^{-}}{{S}_{i}^{+}+ {S}_{i}^{-}}& (\text{i}=\text{1,2}, ...,\text{ n })\end{array}$$

### Statistical analysis

SPSS 25.0 was utilized for statistical analysis to compare variances and conduct the Least Significant Difference (LSD) test. This was done to assess disparities in temperature, soil water storage, crop yield, harvest index, water productivity, and nitrogen use efficiency across different treatments. Mean values between treatments were compared using the LSD test at a significance level of 0.05. The correlation coefficient matrix between different traits was plotted using the R package (corrplot). A correlation coefficient with an absolute value greater than 0.8 between two indicators was considered to have a strong correlation. Geographic images were plotted using ArcGIS 10.2. Histograms and scatter plots were generated using Origin 2023.

## Results

### Soil temperature

**Fig. 3:**
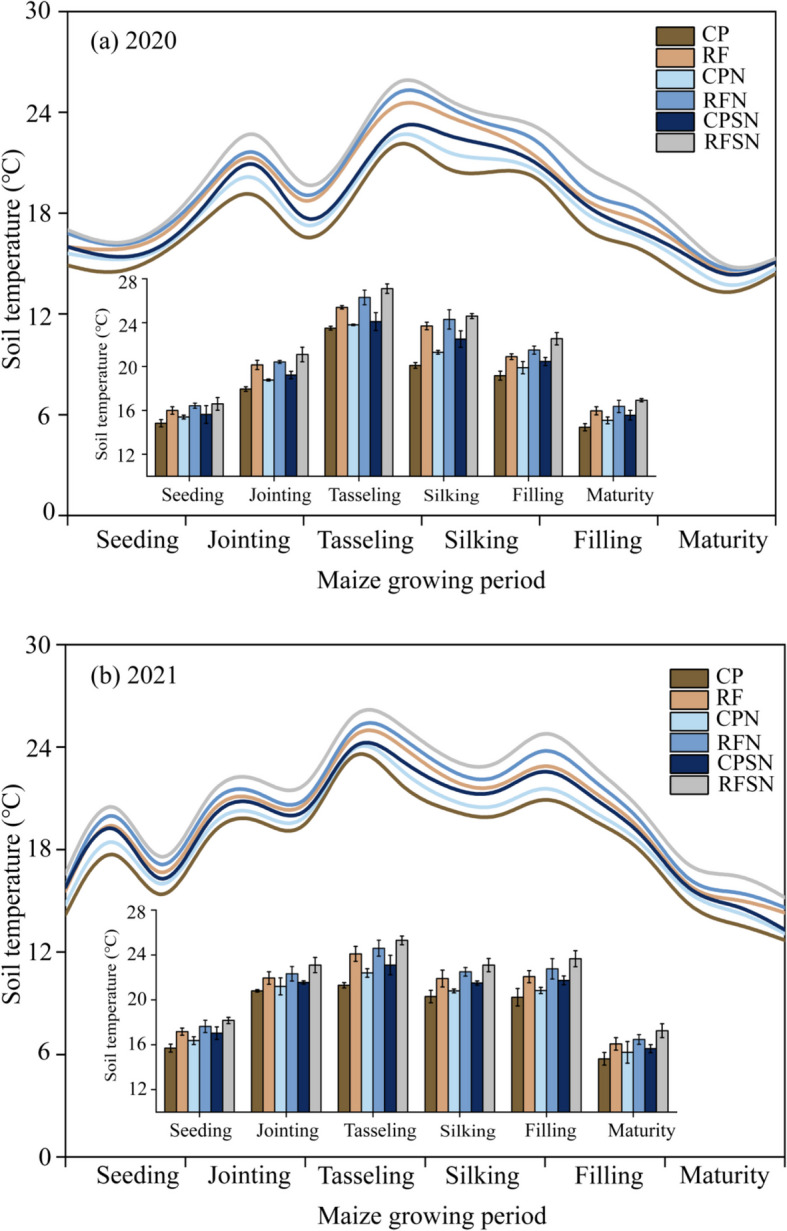
Dynamic changes in soil temperature (0-10 cm) under each treatments during the mazie growing period in 2020 (**a**) and 2021(**b**)

The changes of soil temperature in the 0 -10 cm layer during growth period for each treatment are shown in Fig. 3. Soil temperatures gradually increased after the seedling stage and slowly decreased from the silking stage to maturity (Fig. 3a-b). The range of soil temperature variations during the growing seasons in 2020 and 2021 was 14.2-27.1°C and 13.2-27.3 °C, respectively (Fig. 3). Due to the ridge and furrow rain harvesting planting system covered with film, the soil temperatures of RF were higher 1.3-2.8℃ than that of CP treatment throughout both growing seasons. The findings demonstrate that the ridge and furrow rain-harvesting planting could increased the soil temperature more than the conventional flat planting in consecutive growing periods. During the two experimental periods, RFN and RFSN were increased the soil temperature by 2.36% and 11.02% ,10.4% and 16.83%, 9.09% and 16.83%, 11.11% and 16.04%, 7.84% and 23.52% than that of RF at jointing, tasseling, silking, filling and maturity stages, respectively. Likewise, CPN and CPSN raised soil temperature by 0.25-2.1°C as compared to CP, with significant difference among CP, CPN and CPSN. These trends highlight that nutrient management has a certain impact on soil temperature in 2021 and 2022 (Fig. 3a and b). Consequently, the rankings for soil temperature under various treatments were order: RFSN> RFN > RF > CPSN> CPN> CP, which indicated the planting pattern had large influence on soil temperature.

### Soil water storage

**Fig. 4:**
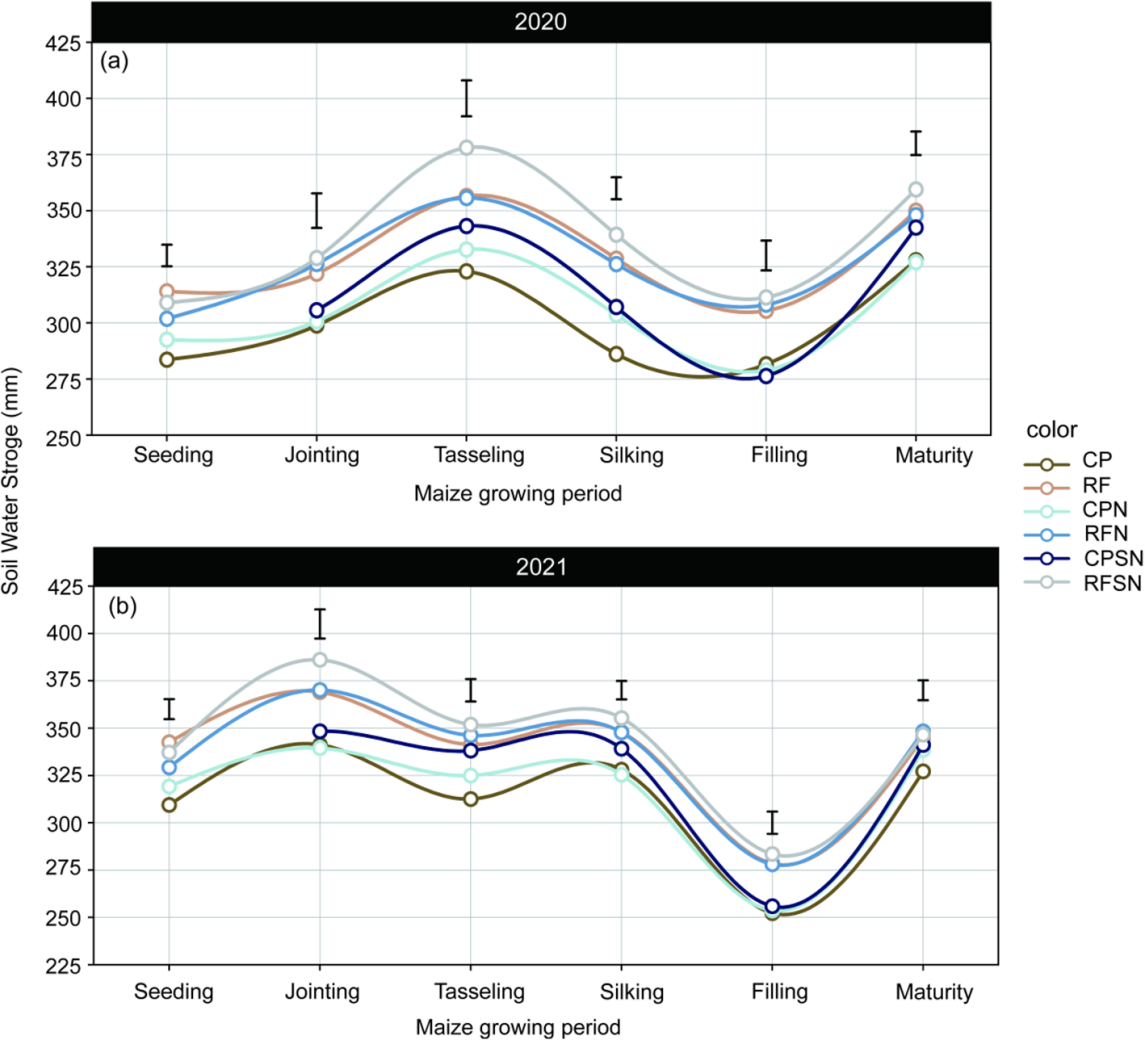
The changes of soil water storage in the 0-60 cm soil depth in planting pattern and integrated nutrient management treatments during the summer maize growing seasons of 2020 and 2021

The dynamics of soil water storage (SWS) varied significantly throughout the six growing periods (Fig. 4). In 2020, the SWS exhibited an increase during the initial growth stage, reaching its peak at the tasseling stage. Subsequently, it decreased as the water consumption of maize increased, reaching its lowest level during the filling stage. However, the water consumption of corn plants gradually decreased and the SWS increased during the maturity period. Moving on to 2021, the SWS showed a gradual rise during the seedling stage and reached its highest level during the jointing stage. Overall, the soil water storage levels (0-60 cm) were higher in 2021 (characterized by wet year) than in 2020 (normal rainfall year). Planting patterns and nutrient management type affected the SWS at 0-60 cm soil layer over the growing seasons, but the effects varied at different growth stages (Fig. 4). The five treatments of RF, CPN, RFN, CPSN and RFSN consistently and significantly enhanced SWS higher than that of CP at tasseling stage by 10.4% (9.2%) , 3% (4%), 10.1% (10.8%), 6.3% (8.2%), and 17.1% (12.6%) in the 0-60 cm layer in 2020 (2021). The ridge and furrow rain harvesting planting system, especially RF, RFN and RFSN significantly improved the SWS by 14.8% (6.1%), 14% (6%), and 18.6% (8.3%) at silking stage, and improved by 8.5% (10.4%), 9.4% (10.2%), and 10.6% (12.4%) at filling stage, and by compared to CP for 2020 (2021). At maturity stage of two years, CP consistently maintained a lower level of SWS, while the highest SWS value of RFSN were increased by the average of 7.8%, 1.6%, 6.1%, 1.4% and 3.3% than in CP, RF, CPN, RFN and RFSN, respectively.

### Grain yield, above-ground biomass and harvest index

**Fig. 5:**
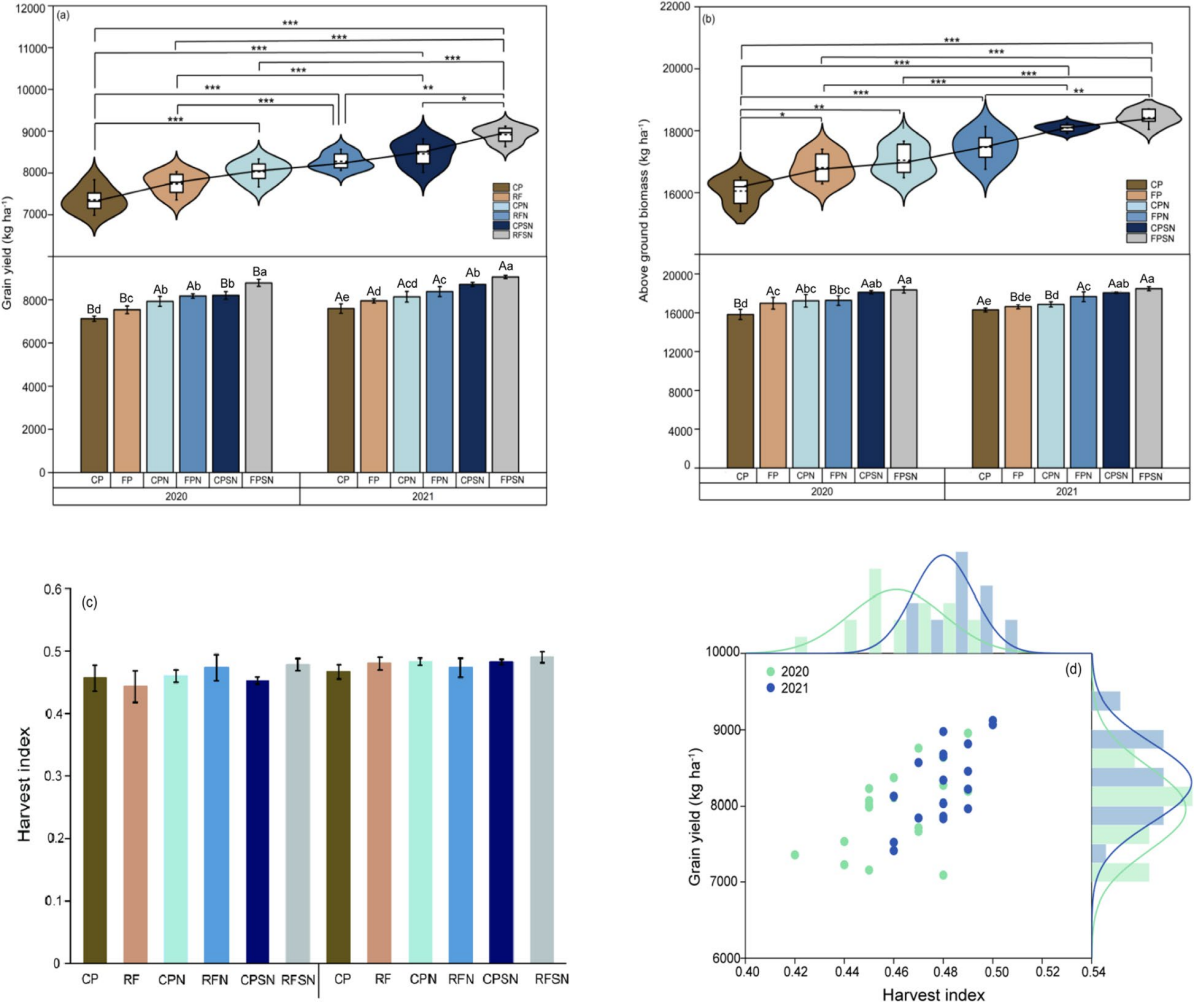
Maize grain yield (**a**), above ground biomass (**b**) and harvest index (**c**) under different treatments in both experimental year and Distribution of grain yield and harvest index of maize (**b**). Bars stand for standard error and different lowercase letters above the bars are significantly different (*P*<0.05). In a and b, the violin plots of grain yield and above ground biomass for maize in this study, solid and dashed lines indicate median and mean, respectively. The box boundaries indicate the upper and lower quartiles, the whisker caps indicate 75th and 25th percentiles. *, ** and *** significant at *P*<0.05, 0.01and 0.001 level, respectively.Various treatments in the same year are indicated by lowercase letters above the columns, and the same treatments across different years are indicated by capital letters above the columns

The maize yield was significantly affected by planting patterns and integrated nutrient management (Fig. 5a). In the 2020 and 2021, the grain yield of RF significantly increased by 4.7% and 5.7% (mean 5.2%) comparing with CP; FPN were increased by 2.9 % and 3.1% (mean 3%) compared with CPN; RFSN were 7.1% and 3.9% (mean 5.5%) higher than that of CPSN. These findings demonstrate that the ridge and furrow rain-harvesting planting treatments significantly increased grain yield compared with the conventional flat planting treatments. In both years, the RFSN were increased the grain yield by 7.4% and 8.1% than that of RFN, with a mean increase of 7.8%; the grain yield of RFN was significantly higher than that of RF by 5.4% and 8.4%, with a mean increase of 6.9% (*P* < 0.05). This suggests that the order of importance for increasing grain yield through nutrient management was integrated nitrogen fertilizer and crop straw > nitrogen fertilizer, as illustrated in Fig. 5a. Regardless of 2020 and 2021, grain yield was highest in FRSN and lowest in CP (Fig. 5a).

Similar trends were observed in above-ground biomass across the treatments over two seasons, with no significant inter-annual variability (Fig. 5b). In 2020 and 2021, the above-ground biomass of the ridge and furrow (RF) planting system was 7.3% and 2.1% (mean 4.7%) higher than that of the conventional flat planting (CP) system (Fig. 5b). Additionally, the average above-ground biomass of the ridge and furrow with nitrogen (RFN) treatments showed a 0.22% and 4.47% higher biomass compared to CPN, with a mean increase of 2.3%. Over the two-year period, the ridge and furrow rain-harvesting planting system consistently exhibited higher above-ground biomass compared to the conventional flat planting treatments. Without considering the planting pattern, the above-ground biomass of RFSN was 6.2% and 3.1% higher than RFN, with a mean difference of 4.6%; RFN increased the above-ground biomass by 1.7% and 7.9% (mean 4.8%) compared to RF. Therefore, the 2 years of above-ground biomass were still highly influenced by ridge and furrow rain-harvesting planting and its interaction with nitrogen fertilizer and crop straw, represented by the high significant effect. The above-ground biomass in all treatments ranked of RFSN>CPSN>RFN> CPN>RF>CP.

Harvest index (HI) under different planting pattern and integrated nutrient management treatments were shown in Fig. 5c and d. The results showed that the HI ranged from 0.43 to 0.51, with an average of 0.46 and 0.48 in 2020 and 2021, and most of HI within 0.45-0.5 (Fig. 5c). In the same growth period, the highest harvest index in RFSN treatment was 51.1% and 56.9% higher than that of CP in 2021 and 2022. The lowest harvest index is CP in 2020 and 2021. However, no significant differences were found among RF, RFN, CPN, and CPSN in both years.

### Grain N content, straw N content, plant N uptake in maize

Nitrogen content of the grain and straw was strongly influenced by ridge and furrow rain-harvesting planting system and nutrient management in these two years (*P* <0.05, Table 1). Across all years, RFSN had the greatest grain N content and straw N content, which were 18.1% and 7.13% higher than CPSN in 2020 and by17.7% and 4.1 % in 2021, respectively. The average N content of the grain and straw of FPN was 40.1% (10.1%) and 23% (11.2%) lower than that of FPSN in 2020 (2021), respectively. CP had the lowest average N content of the grain, while which had the lowest at N content in straw. The N contents of the both grain and straw were highest in RFSN, followed by CPSN, FPN and CPN, but CP had the lowest N content of the grain and straw in both years (Table 1).

**Table 1:** Grain N content and Straw N content under different planting pattern and integrated nutrient management treatments in maize growing periods

Year	Treatments	Grain N content (g kg^-1^)	Straw N content (g kg^-1^)
2020	CP	7.83e ± 0.60	5.35d ± 0.45
	RF	8.22e ± 0.66	5.96c ± 0.25
	CPN	9.15d ± 0.12	7.02c ± 0.31
	RFN	10.04c ± 0.37	7.31b ±0.42
	CPSN	11.91b ± 0.26	7.52b ± 0.33
	RFSN	14.07a ± 0.22	8.06a ± 0.03
2021	CP	7.82d ± 0.43	6.23c ± 0.20
	RF	9.30c ± 0.31	6.38c ± 0.43
	CPN	9.55b ± 0.10	8.03b ± 0.08
	RFN	10.37b ± 0.32	8.20b ± 0.27
	CPSN	10.83ab ± 0.89	8.76a ± 0.49
	RFSN	12.76a ± 0.22	9.12a ± 0.12

**Fig. 6:**
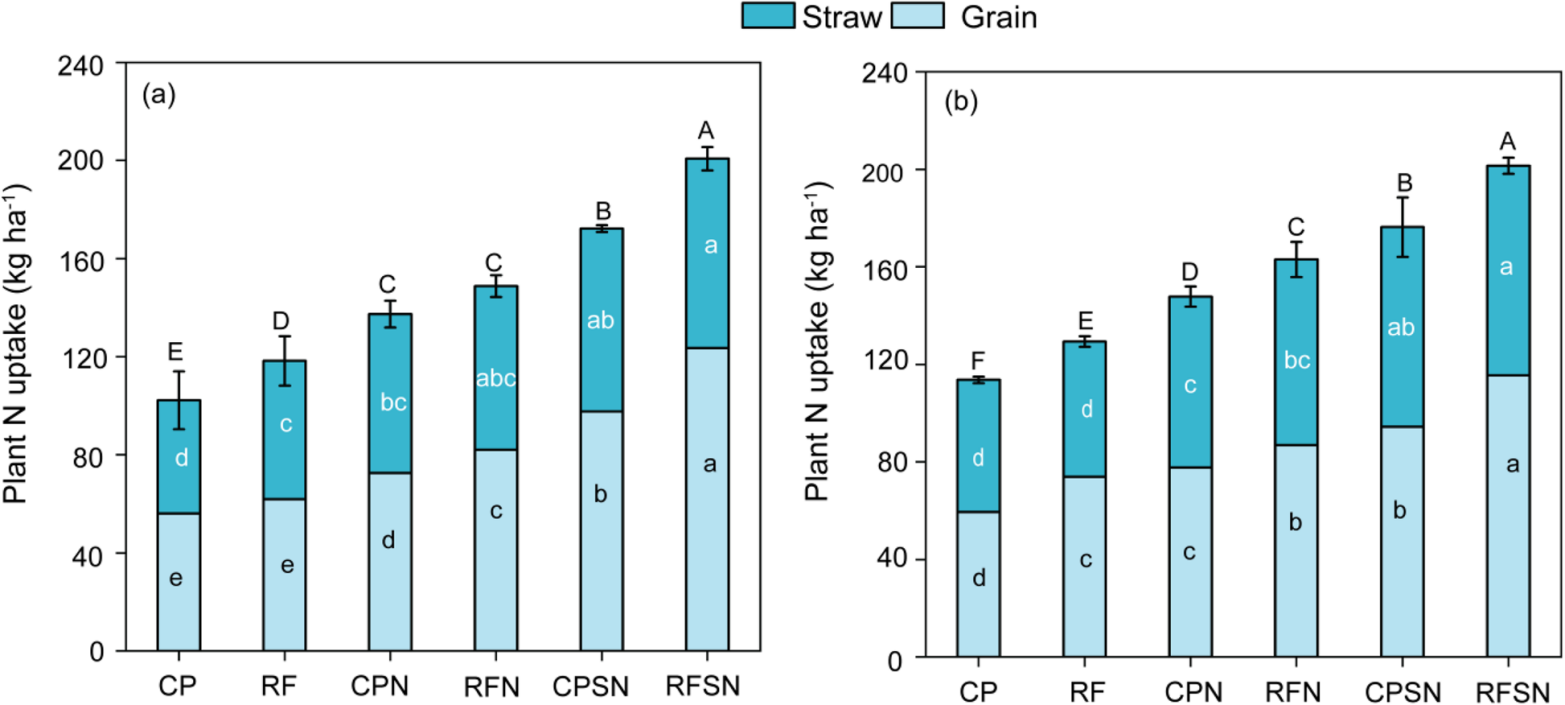
Grain N uptake and straw N uptake under different planting pattern and integrated nutrient management in 2021 (**a**) and 2022 (**b**). Different letters above the bars indicate a significant difference at *P* < 0.05 according to the Duncan test

According to Fig. 6, RFSN treatments significantly increased the plant N uptake of maize from 2020 to 2021. In all treatments, the plant N uptake in different organs was ranked as grain > straw. In 2020 and 2021, the plant N uptake in RFN was 148.8 kg ha^-1^ and 163 kg ha^-1^), which was 25.7% and 25.9% higher than that of RF (Fig. 6). Furthermore, the CPN showed an improvement in plant N uptake by 34.4% in 2020 and by 30% in 2021 compared to the CP. It is worth noting that both FPN and CPN treatments resulted in an initial increase in plant N uptake in 2020 and 2021 due to the application of N fertilizer. Additionally, RFSN improved plant N uptake than that in RFN by 34.9% in 2020, and 23.5% in 2021, respectively; CPSN increased the plant N uptake by 25.3% and 19.2% than that of CPN. These results indicated that the improvement in plant N uptake was mainly associated with the regulation of straw and fertilizer, which was promoted by the increase in soil moisture and temperature caused by ridge and furrow rain-harvesting planting system practices.

### Water productivity (WP) and N use efficiencies

**Fig. 7:**
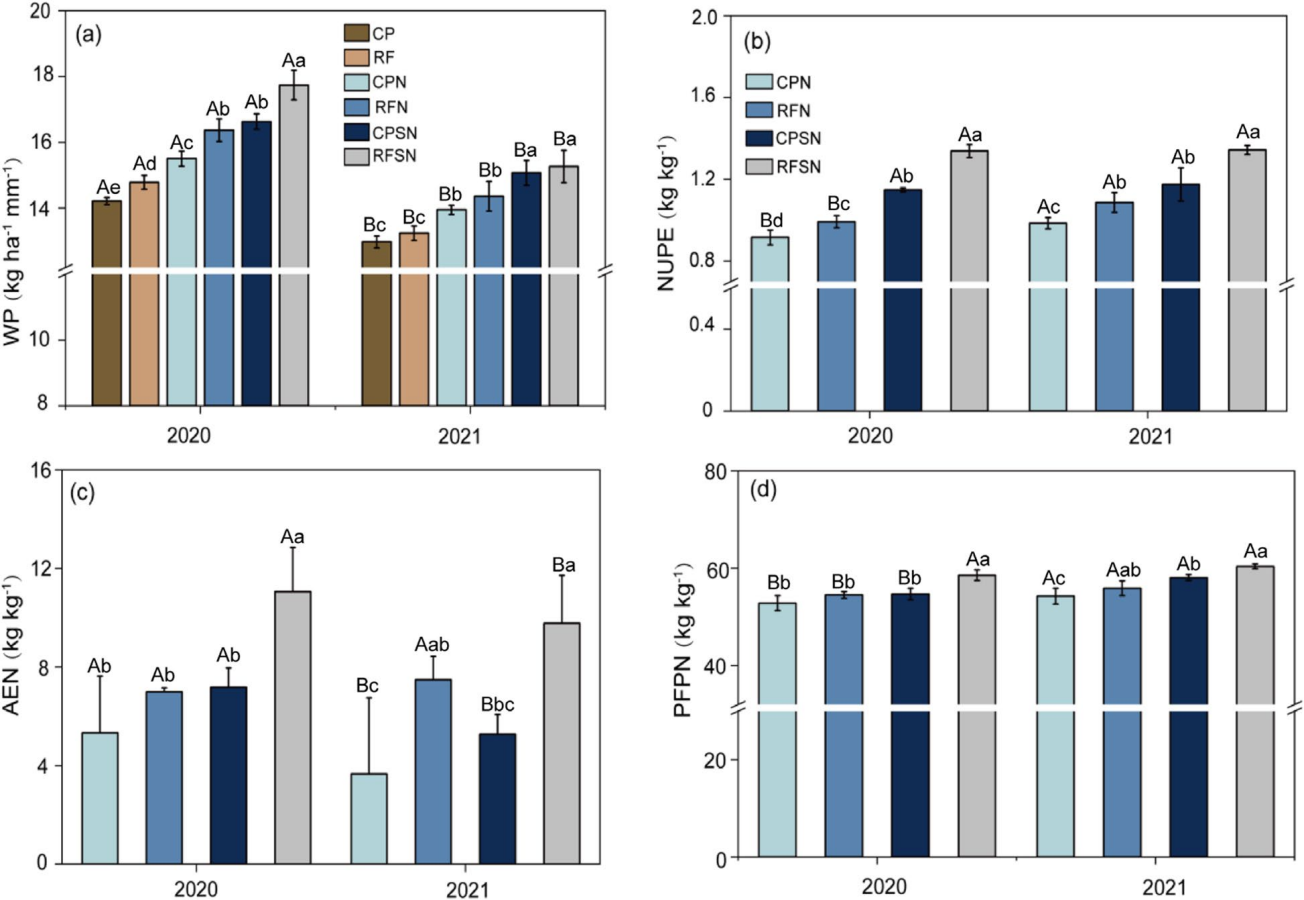
Water productivity (WP), N uptake efficiency (NUPE), agronomy efficiency of N (AEN), and partial factor productivity of N fertilizer (PFPN) under different treatments in 2020 and 2021.Various treatments in the same year are indicated by lowercase letters above the columns, and the same treatments across different years are indicated by capital letters above the columns

Under the same nutrient management, ridge and furrow rain-harvesting planting system treatments significantly increased WP compared with the conventional flat planting treatments (Fig. 7a). In 2020 and 2021, FP were increased by 4.01% and 2.03 % (mean 3.02%) compared with CP. RFN were 5.5% and 2.9% (mean 4.2%) higher than that of CPN. Moreover, the RFSN showed an improvement in WP by 6.6% in 2020 and by 1.26% in 2021 compared to the CPSN. However, there were difference of the WP in various nutrient management treatments under same planting pattern (Fig. 7a). In both years, the RFSN were increased the WP by 8.3% and 6.3% (mean 7.3%) than that of RFN; the WP of RFN was significantly higher than that of RF by 10.7% and 8.5% (mean 9.6%), respectively (*P* < 0.05), indicating the nutrient management significantly effect WP were showed crop straw combine with nitrogen fertilizer>nitrogen fertilizer. Regardless of 2020 and 2021, the WP effects followed the order of RFSN>CPSN>RFN>CPN>FR>CP.

Planting pattern and integrated nutrient management significantly influenced maize NUPE in both years (Fig. 7b). The RFN improved NUPE by 8.3% and 10.2% (mean 9.3%) over CPN, while RFSN enhanced NUPE by 16.5% and 14.3% (mean 15.4%) over CPSN in 2020 and 2021. NUPE was higher in the ridge and furrow rain-harvesting planting system compared to conventional flat planting treatments. In both years, the RFSN were increased the NUPE by 34.9% and 23.5% (mean 29.2%) than that of RFN, respectively (*P* < 0.05). The NUPE of CPSN were significantly higher by 25.3% and 19.2% than CPN, with a mean increase of 22.3%. These results indicating NUPE showed a gradual increase with the integrated of nitrogen fertilizer and straw management under ridge and furrow rain-harvesting planting system. Over the two years, RFSN had the highest NUPE, resulting in the following order: RFSN > CPSN > RFN > CPN.

Agronomic Efficiency of N (AEN) is a metric used to quantify the impact of nitrogen application on yield increase. Significant differences (*p*<0.05) were observed in AEN among various treatments, The RFN significantly increased AEN than that of CPN, with a mean increase by 67.8% , whereas the AEN of RFSN was 53.9% and 85.2% higher than that of CPSN in 2020 and 2021, with a increase of 69.6%. These results indicated that the ridge and furrow rain-harvesting planting system and nutrient management playing a strong influencing role. Over two growing seasons, the RFSN treatment exhibited the highest AEN values, showing increases of 58 % and 31% compared to RFN (Fig. 7c), with a increase of 44.5%. Similarly, CPSN treatment showed AEN increases of 34.7% and 43.9% (mean 39.3%) compared to CPN in 2020 and 2021, respectively. These findings suggest that integrated of nitrogen fertilizer and straw management significantly enhances AEN under ridge and furrow rain-harvesting planting system.

In two years, planting patterns and integrated nutrient management had significant effects on PFPN (Fig. 7d). Comparing with CPN, the PFPN of RFN significantly increased by 3.2% and 2.9% (mean 3.1%) in 2020 and 2021, respectively (Fig. 7d). The PFPN of RFSN was 7.1% and 3.9% (mean 5.5%) higher than that of CPSN in both years. These findings demonstrate that the ridge and furrow rain-harvesting planting treatments significantly increased grain yield compared with the conventional flat planting treatments. In both years, the RFSN were increased the PFPN by 7.4% and 8.1% than that of RFN, with a mean increase of 7.8%; the PFPN of CPSN was significantly higher than that of CPN by 3.5% and 7.1%, with a mean increase of 5.3% (*P* < 0.05). This suggests that the order of importance for increasing PFPN through nutrient management was integrated nitrogen fertilizer and crop straw > nitrogen fertilizer. The FPFN were highest in RFSN, followed by CPSN, FPN, but CPN had the lowest FPFN in both years (Fig. 7d).

### Soil organic carbon and nitrogen fractions

**Fig. 8:**
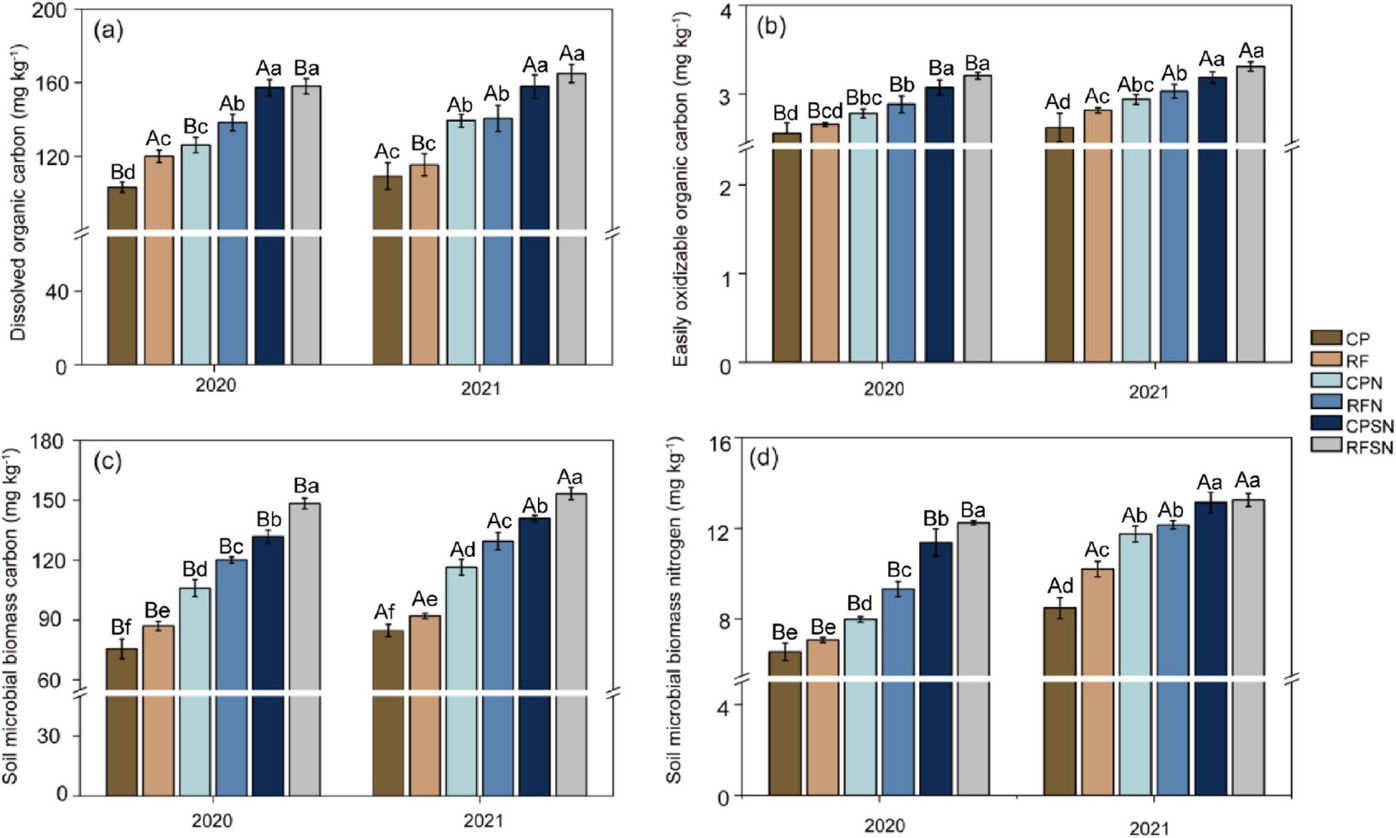
Dissolved organic carbon (DOC), easily oxidizable organic carbon (EOC), soil microbial biomass carbon (MBC), and soil microbial biomass nitrogen (MBN) under the various planting pattern and integrated nutrient management treatments in 2020 and 2021. Various treatments in the same year are indicated by lowercase letters above the columns, and the same treatments across different years are indicated by capital letters above the columns

To clarify the effect of planting pattern and integrated nutrient management on the distribution of SOC fractions, the contents of the four soil organic carbon and nitrogen fractions (DOC, EOC, MBC and MBN) were determined in the 0-20 cm soil layer with similar trends: RFSN>CPSN>RFN>CPN>RF>CP (Fig. 8a-d). The DOC concentrations of the RF, CPN, RFN, CPSN, and RFSN treatments were significantly higher than that in CP by 16.3%, 22.3 %, 34.1%, 52.5 % and 53.2% in 2020, and by 5.6%, 27.6%, 28.7%, 44.6%, and 51.1% in 2021, respectively (Fig. 8a). The EOC concentration was significantly higher by 4.0%-25.4 % (2020) and 7.3%-26.1% (2021) in RFSN compared to the other treatments, and there was a slight increase in EOC concentration corresponding to the input of straw and nitrogen fertilizer to the soil. A significant difference was found between the CPSN, RFN, CPN and RF treatments in both years (Fig. 8b).

Particularly in RFSN, increased the concentrations of MBC in the 0-20 cm layer by 12.5% (8.8% ) and 96.3% (80.8%) compared to CPSN and CP in 2020 (2021) (Fig. 8c). The lowest MBC concentration value was found in the CP treatment, the MBC values increased by 74.3% (66.1%) , 58.8% (52.6%), 40.2% (37.3%), and 15.1% (8.5%) in the CPSN RFN, CPN, and RF treatments in 2020 (2021), respectively (Fig. 8c). The MBN were stimulated by 86.7%, 73.5%, 42.1%, 21.8%, and 8.0% under RFSN, CPSN, RFN, CPN, and RF relative to CP in 2020, and by 56.2%, 54.9%, 43.2%, 38.4%, and 20.3% in 2021, respectively (Fig. 8d).

### Correlation among variables and principal component analysis

**Fig. 9:**
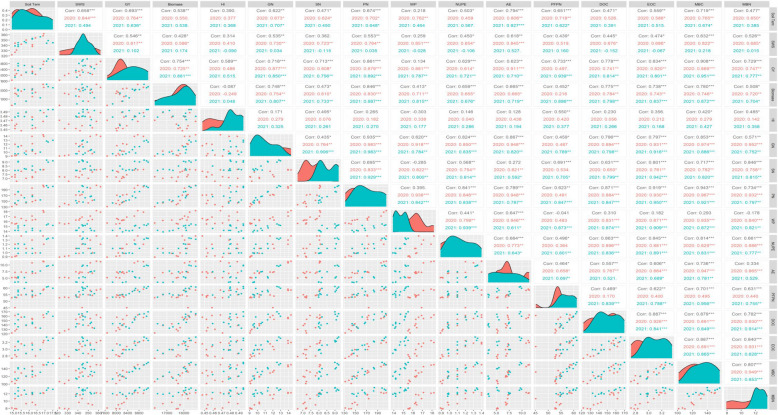
Correlation relationship among soil temperature (Soil TEM), soil water storage (SWS), grain yield (GY), HI, grain N content (GN), straw N content (SN), plant N uptake (PN), water productivity (WP), N uptake efficiency (NUPE), agronomy efficiency of N (AEN), and partial factor productivity of N fertilizer (PFPN), dissolved organic carbon (DOC), easily oxidizable organic carbon (EOC), soil microbial biomass carbon (MBC), and soil microbial biomass nitrogen (MBN) in 2020 and 2021

In order to determine the key factors that affected soil hydrothermal environment and yield components, the correlations between soil temperature in 0-10cm soil layer at maturity stages, SWS, grain yield, above-ground biomass, HI, grain and straw N content, plant N uptake, WP, NUPE, AEN, PFPN, DOC, EOC, MBC and MBN during different growth periods (Fig. 9). Strong positive correlations coefficients (r≥0.71) existed between the grain and straw N content, plant N uptake, DOC, EOC, MBC and MBN were significantly positively correlated with grain yield, while there were significant correlations with the above-ground biomass had significant correlations with grain N content, plant N uptake, DOC, EOC and MBC (*r*=0.748, 0.846, 0.778, 0.843 and 0.78) (Fig. 9). Grain N content was significantly related to plant N uptake, NUPE and AEN, and the correlation coefficients ranged from 0.824 to 0.935 (Fig. 9). In particular, straw N content (*r*=0.801, 0.717 and 0.846 ) and plant N uptake (*r*=0.871, 0.919, and 0.903) was positive correlated with DOC, EOC and MBC (Fig. 9).

**Fig. 10:**
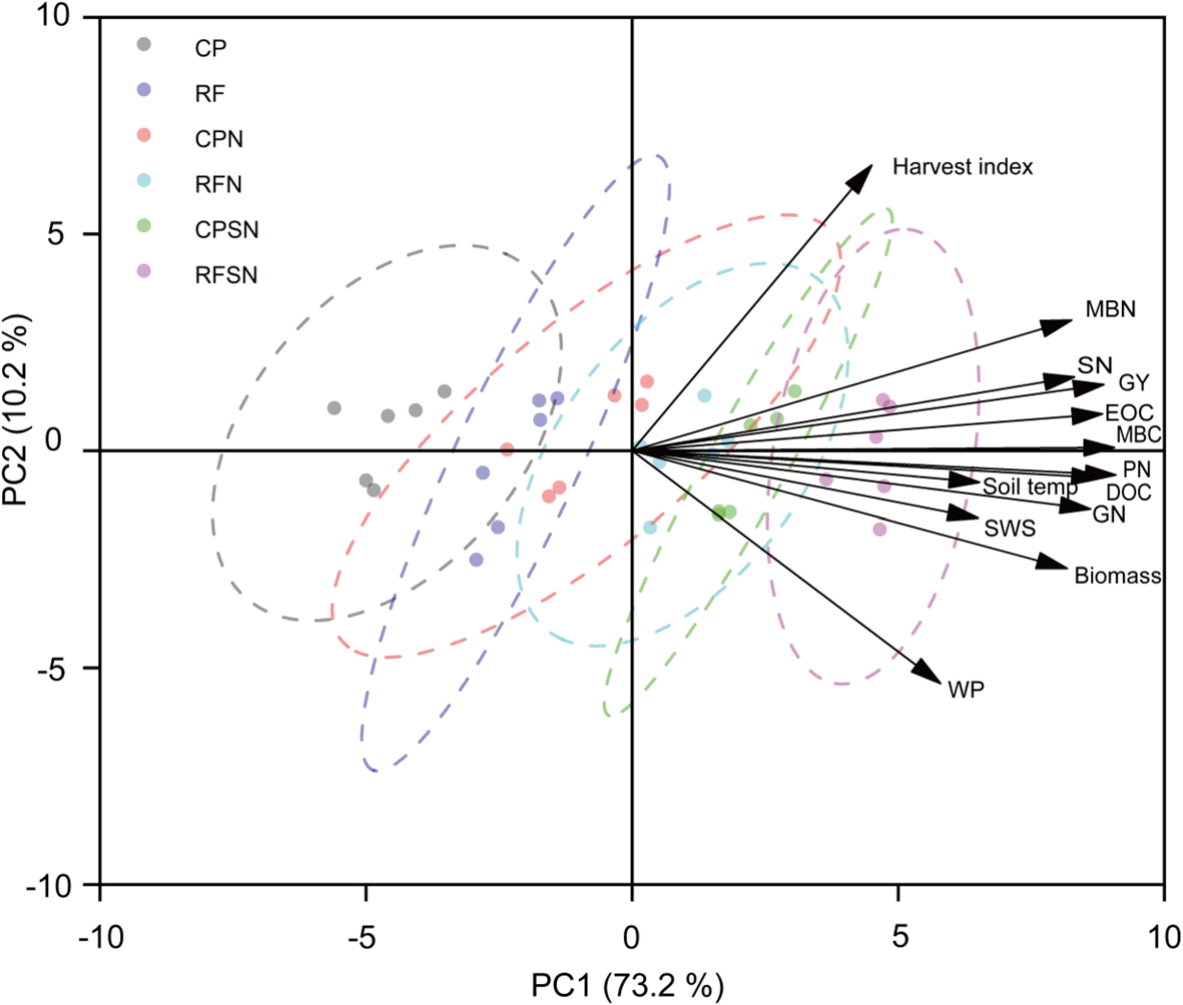
Principal component analysis of soil temperature (Soil Temp) in 0-10cm soil layer at maturity stages, Soil water storage (SWS) in 0-60 cm soil layer at maturity stage, grain yield (GY), above-ground biomass, Harvest index, grain N content (GN) , straw N content (SN), plant N uptake (PN), water productivity (WP) , dissolved organic carbon (DOC), easily oxidizable organic carbon (EOC), soil microbial biomass carbon (MBC), and soil microbial biomass nitrogen (MBN)

The PCA biplot (Fig. 10) represents the correlation between soil hydrothermal environment and yield components under planting pattern and integrated nutrient management, and the cosine value between each pair of black arrows represents the correlation between the two variables. The first principal component (PC1) could explain 73.2% variability, while the PC2 was associated with 10.2% variability. Grain yield, straw N content, plant N uptake, grain N content, above-ground biomass, soil temperature, SWS, DOC, EOC, MBC and MBN had positively correlated. Different treatments showed good differentiation on PC1 (Fig. 10).

### Determination of optimal management pattern based on gray relation analysis and entropy weighted TOPSIS model

The pursuit of increasing maize yield is a global goal in agriculture, with cropping patterns and nutrient management playing crucial roles in sustainable agricultural development. Planting and fertilization strategies for maize differ based on specific indicators. Hence, it is essential to develop a comprehensive evaluation model that considers each indicator individually to objectively and accurately assess maize indicators. The ultimate aim of this evaluation is to determine the optimal combination of planting patterns and nutrient management treatments. The study focused on analyzing grain yield, above-ground biomass, harvest index (HI), grain and straw nitrogen (N) content, plant N uptake, water productivity (WP) using gray relation analysis and entropy weighted TOPSIS model to assess the overall performance of maize (Table 2). The average correlation coefficients between the rated value of gray relation analysis model and another model ranged from 0.934 ~ 0.970 in 2021 to 0.817 ~ 0.939 in 2022 (Table 2). The results indicate that there are slight differences in the results and rankings of the same treatment under different integrated evaluation methods.The greater the evaluation value, the better the evaluation object. Obviously, CP is not the best combination. This study proposed six planting models and nutrient treatments, the better combinations obtained from this evaluation were consistent: FPSN was rank first, follow by CPSN in 2020 and 2021 (Table 2). Specifically, the best treatment was ridge and furrow rain harvesting planting system and integrated straw and nitrogen management, which could achieve high yield while maintaining a high level of water-N use efficiency.

**Table 2:** Results of correlation degree and ranking based on gray relation analysis and entropy weighted TOPSIS evaluation model

Gray relation analysis					Entropy weighted TOPSIS evaluation model			
Treatment	2020 values	Rank	2021 values	Rank	2020	Rank	2021	Rank
CP	0.906	5	0.903	6	0	6	0	6
RF	0.9	6	0.924	5	0.303	5	0.19	5
CPN	0.917	4	0.928	4	0.466	4	0.36	4
RFN	0.938	3	0.928	3	0.581	3	0.585	3
CPSN	0.943	2	0.951	2	0.741	2	0.78	2
RFSN	0.986	1	0.997	1	1	1	0.999	1

## Discussion

### Planting pattern and integrated nutrient management effects on soil temperature

This study investigated the impact of planting pattern and integrated nutrient management on soil hydrothermal environment, yield and use efficiency. Previous research has primarily focused on the interaction between indicators under different film mulching with the uniform planting patterns [17] or different planting patterns and N fertilizer rates [18], or different ridge-furrow ratios with the same planting pattern [5] , or variable planting patterns with the same organic amendments [19]. Our study uniquely explored the specific effects of N fertilizer or crop straw in grouping different integrated nutrient management measures with varying planting pattern on maize production soil hydrothermal environment, yield and use efficiency.In present study, soil temperatures showed gradually increased after the seedling stage and slowly decreased from the silking stage to maturity (Fig. 3a-b). Vaccari et al. [20] reported that soil temperature undergoes daily and seasonal changes, which are shaped by variables such as ambient air temperature, solar radiation, and the transfer of energy between the soil and the atmosphere. Our findings also revealed that the significant impact of the chosen planting pattern has a substantial impact on soil temperature, with the rankings for soil temperature in the 0-10 cm layer during the growth period under different treatments as RFSN>RFN>RF>CPSN>CPN>CP (Fig. 3). Unlike other yield and efficiency indicators, integrated nutrient management had a certain effect on soil temperature, and the planting pattern had largely increased the soil temperature, particularly in the ridge and furrow rain-harvesting planting treatment. The implementation of mulching in the ridge and furrow rain-harvesting planting system can effectively reduce the long-wave solar radiation emitted from the soil to the atmosphere, and thus slowed down the loss of soil heat and increased the thermal environment of the farmland soil [21]. Previous study have reported that the soil temperature of 0-100cm layer with the ridge and furrow rain-harvesting planting system treatment was consistently 1.0~3.07 ℃ higher compared to conventional flat planting during the jointing, flowering, filling, and harvesting stages [22]. This temperature difference significantly enhanced maize growth and development at various stages. We also obtained the results in present study that the ridge and furrow rain harvesting planting system covered with film, the soil temperatures of RF were higher 1.3-2.8℃ than those of CP treatments throughout both growing seasons (Fig. 3). further confirmed that the mulching can effectively increase soil temperature, while ridge and ridge mulching has a greater effect on increasing soil temperature. This phenomenon has been validated in prior research, demonstrating that plant canopies are sufficiently small for solar radiation to penetrate and heat the surface soil beneath the plastic film. A previous study also reported a similar increase in soil temperature with the use of continuous furrow and plastic film covering in winter rapeseed fields in northwest China [23]. Li et al.[24] found that the ridge-furrow with film mulching increased soil temperature ranged from 6.7% to 20.8% at 50-150 mm soil layers compared to the control, respectively. The ridge and furrow rain-harvesting planting treatment not only increased planting surface area and ground roughness, and improved the capacity to absorb solar radiation, but also provided a warming effect through ground film. The highest values of soil temperature were observed for RFSN at the whole maize growing stages (Fig. 3). Recently, the impact of crop straw addition on soil temperature can be explained by the combined effect of factors such as soil surface reflectivity, thermal conductivity and albedo changes. Therefore, the ridges treated with N fertilizer and straw have been shown to moderate soil temperature fluctuations and warm the soil by decreasing soil surface albedo and thermal conductivity at ridge tops. However, it is important to note that this treatment does have an impact on soil temperature at furrow bottom.

### Effect of planting pattern and integrated nutrient management on soil water storage

Soil moisture is primarily influenced by rainfall infiltration, with crops absorbing and utilizing only about 25% of the water, while the remaining 65% is lost to evaporation and runoff [25]. Substantial fluctuations in SWS due to varying inter-annual precipitation. In our current study, we identified the SWS levels (0-60 cm) were higher in 2021 (characterized by wet year) than in 2020 (low annual precipitation) (Fig. 4). Previous studies have shown that soil moisture is closely relevant to the temporal distribution and amount of rainfall, planting pattern, and nutrient application [26–28]. During the seeding stages, the SWS in the FP, FPN and exhibited a significant increase compared to the conventional flat planting treatment (Fig. 4 ). This can be attributed to the bits of soil evaporation in the initial growth stage of plants. Specially, when the crop canopy is small and not yet dense, the use of ridge and furrow rain-harvesting planting plays a crucial role in reducing soil evaporation [29]. In addition, when corn is growing vigorously, water gradually evaporates from the soil in the middle and late stages and is converted into transpiration within the plant . Present studies have demonstrated that the ridge and furrow rain-harvesting planting can effectively improve the maize growth rate, which can lead to an increase in the transpiration rate of the plant, which may help counterbalance the rise in soil moisture resulting from flat land and ridge and furrow rain-harvesting planting, which caused the SWS decreased in lately growth stage [30].

In the current study, we identified the FPSN and CPSN increased soil water storage (*P* < 0.05) at all stages, compared to FPN and CPN (Fig. 4). The results further indicated that straw management positively soil water storage regardless of the planting pattern. This effect can be attributed to the ability of straw to retain water for a longer period, resulting in slower surface water flow and increased water infiltration into the soil. The addition of straw also improves water infiltration by modifying soil physical properties through the presence of organic matter and providing additional pathways for water absorption [31, 32]. Previous studies have demonstrated that straw returning and different planting patterns play a significant role in maintaining soil water storage through various mechanisms [33].

### Planting pattern and integrated nutrient management effects on grain yield performance and above-ground biomass

Planting pattern and nutrient management are critical factors influencing maize growth. In present study, we found that the ridge and furrow rain-harvesting planting system treatments significantly increased grain yield and above-ground biomass compared with the conventional flat planting treatments. The adoption of furrow and ridge rainfall harvesting pattern planting pattern can be attributed to enhanced water availability and temperature condition, leading to a substantial increase in grain yield and biomass (Fig. 5a), consistent with findings from previous studies on maize, potato [34], soybean [35], millet [36]. The furrow and ridge rainwater harvesting planting system is effective in reducing soil evaporation by using a physical barrier of plastic film [37]. Additionally, the topography of the ridges helps direct rainwater into the furrows, and allowing the collected precipitation to seep into deeper soils, which increased water productivity and grain yields, particularly in drought conditions. Previous studies have confirmed that mulching in the furrow and ridge rainwater harvesting planting system suppressed weed competition for resources and enhanced crop growth, which led to a significant increase in grain yield and above-ground biomass [38]. Similarly, Fan et al. [28] found that the 8-year average maize yield increased by 22.7% in the ridge and furrow rain harvesting planting system most significantly compared with the conventional flat planting treatments. Fang et al. [39] found that the wheat yield under the ridge and furrow rain-harvesting planting system increased by 13.0-32.9%, 15.5%-35.2% and 27.2-58.9% in 2013-2014, 2014-2015 and 2015-2016. Mo et al. [14] showed the grain yield and above-ground biomass in RFRH increased by 6.8%-73.1% and 70.9%-87.2% compared with the conventional flat planting treatments. Interestingly, we found that the grain yield and above-ground biomass of RFN was significantly increased by a mean of 6.9% and 4.8% than that of RF, respectively (Fig. 7a and b). The present results also shown that this result suggests that nitrogen fertilizer resulted in increase of grain yield, and a possible synergistic effect of N and water on yield performance, which has been previously reported [40]. Besides, our study found the RFSN were highest in the grain yield and above-ground biomass, with a increase of 7.8% and 4.6 % than that of RFN (Fig. 7a and b). These results shown that grain yield and above- ground biomass were still highly influenced by nitrogen fertilizer and its interaction with crop straw treatments. Previous studies by Alburquerque et al. [41] also highlighted the positive effect of straw addition on crop production, attributing it to the retention of nutrients, particularly beneficial in rough and barren soils. Ridge and furrow rain-harvesting planting system and nitrogen fertilizer management is effective in optimizes the water, nutrient and soil environmental factors necessary for crop growth. Specifically, mulching combined with integrated of nitrogen fertilizer and crop straw resulted in a significant increase in production.

### Planting pattern and integrated nutrient management effects on water productivity

Crop production in arid and semi-arid regions has faced challenges of limited water availability. It is critical to develop cropping patterns that conserve water, maintain economic yield and increase maize production. The primary goal is to improve water productivity (WP) by reducing crop evapotranspiration (ET) while increasing grain yields [42]. The findings in this study suggest that the ridge and furrow rain-harvesting planting system treatments significantly increased WP compared with the conventional flat planting treatments (Fig. 7a). This is mainly due to the fact that ridge and furrow rain-harvesting planting system enhances the hydrothermal environment by using mulching, and reduced ineffective soil evaporation with larger canopies and enhances water productivity through effective rainwater harvesting [7]. These alterations promote root system growth and extension, and improving the crop's capacity to absorb water and nutrients. Additionally, this planting pattern reduces soil moisture evaporation through crop transpiration, and ultimately enhancing the WP. Previous studies have shown similar results in line with previous studies conducted in semi-arid Kenya. Gu et al. [23] found that the ridge and furrow rain-harvesting planting system treatments was increased the WP by 41.8 and 93.1% than conventional flat planting treatments, respectively. However, In this study, the WP of FPN was significantly higher than that of RF, while the WP of RFSN was the highest (Fig. 7a). These results indicated that ridge and furrow rain-harvesting planting system and nutrient management could significantly improve water utilization. Previous studies have also shown that the addition of biochar under ridge and furrow rain-harvesting systems increased the WP and NUE by 14.5% and 12.0% [37]. This may be due to the fact that the addition of crop straw increased the saturated water content of the soil, higher soil moisture and nitrogen effectiveness provided an important guarantee of high yields, plus the availability of nutrients in the root zone, and the good water supply under the rain-flooding mode. This may be due to the fact that the addition of organic matter increased the saturated water content of the soil [43], which helps in crop growth, yield formation and water and fertilizer utilization efficiency. More importantly, efficient utilization of precipitation resources in semi-arid zones is a key factor influencing yield stability in maize [7]. Within a certain range, nutrient management can promote crop growth and facilitate water absorption and utilization by the root system, and ultimately increased WP promoted the uptake of nutrients by the crop.

### Planting pattern and integrated nutrient management effect on nitrogen utilization

Effective water-saving planting patterns can play a crucial role in determining the availability of nutrients in the soil by influencing the conversion of existing nutrients through moisture and facilitating the conversion of unavailable nutrients into forms that can be easily absorbed by plants [44]. In addition, water also affects the conversion rate of nutrients in the soil, and therefore implementing the ridge and furrow rain harvesting system is an important measure to increase soil moisture, which can significantly enhance nitrogen uptake and utilization by crop [45]. Our study results indicated that the ridge and furrow rain-harvesting planting system increased the plant N uptake (Fig. 7a and b) and effectively promotes nitrogen utilization (Fig. 7b, c and d). Previous research findings have demonstrated that the ridge-furrow mulching cultivation could efficiently enhance nitrogen accumulation in maize, leading to a 49.6% increase in PFP and a 25.7% increase in AEN. Our findings further revealed that the RFN improved the NUPE, AEN and FPFN compared with CPN, with a mean increase of 9.3%, 67.8% and 3.1% (Fig. 7b, c and d), whereas the NUPE, AEN and FPFN of RFSN treatment significantly increased compared to CPSN, with a mean increase of 16.5%, 69.6% and 5.5% in both growing years (Fig. 7b, c and d). Overall, these results highlight the NUPE, AEN and PFPN was higher in the ridge and furrow rain-harvesting planting system compared to conventional flat planting treatments. These results indicated that ridge and furrow rain-harvesting planting system effectively improves soil water temperature, regulates crop root distribution, and creates a favorable rhizo-sphere microenvironment. These factors provide optimal conditions for crop nutrient absorption, facilitating nitrogen accumulation and utilization, and are closely related to NUPE, AEN, and PFPN, which are influenced by soil nutrient availability and soil water status. Our study also found that the RFSN were significantly improvement in the NUPE, AEN, and FPFN than that of RFN, with a mean increase of 29.2%, 44.5% and 7.8%. whereas CPSN increased NUPE, AEN and PFPN than that of CPN, with a mean improvement by 22.3%, 39.3% and 5.3% (Fig. 7 b-d). These results indicated effective nutrient management can also enhance NUPE, AEN, and PFPN..

### Integrated decision and evaluation based on gray relation analysis and entropy weighted TOPSIS model

Many Researches investigated the impact of furrow and ridge rainwater harvesting patterns with biochar or fertilizer on maize yield, water and fertilizer use efficiency, soil temperature, and crop quality. Due to the diverse water and nutrient requirements across different parameters, relying solely on qualitative analyses is inadequate for research goals. Therefore, this study adopts gray relation analysis and entropy weighted TOPSIS model to make the evaluation more objective and reliable. Gray relation analysis is a method of quantitative comparison using mathematical means and comparability and similarity of series. The traditional TOPSIS model generally adopts hierarchical analysis or expert opinion survey method when determining the weighting factors of evaluation indicators. However, entropy weighted TOPSIS model carries out dimensionless treatment on all the index traits by vector normalization method, which can compare the research objects under the same criteria for evaluation, avoid the influence of subjective factors to a certain extent, and evaluate the traits under different treatments more accurately. At present, gray relation analysis and entropy weighted TOPSIS model methods have been widely used. Zhang et al. [12] adopted the membership function, principal component analysis, gray relation analysis and entropy weighted TOPSIS model were used to evaluate the best irrigation amount and fertilization rate, and finally determined that 270-290 mm (irrigation) and 167-193 kg N ha^-1^ deserves further development and validation for improving potato yield. Wang et al. [48] was studied the irrigation and N fertilization on fruit yield, quality, net income and water-N productivity on cherry tomato and found that the optimization of irrigation of 80% ET0 and 360 kg N ha^-1^ N fertilizer supply schedule based on the entropy-weight TOPSIS method was conducive to maintaining yield and income better. In this study, both models can determine the weight of indicators without being affected by personal factors. The results of this study showed that RFSN treatment (application of maize straw 1.5 t ha^-1^ and chemical nitrogen fertilizer of 180 kg ha^-1^ in ridge and furrow rainwater harvesting system) was the best combination management for maize cultivation.

## Conclusions

Ridge and furrow rain-harvesting planting system and integrated nutrient management had significantly effects on soil temperature, gravimetric soil water content, soil water storage, grain yield, above-ground biomass, N content, plant N uptake, WP, NUPE, AEN and PFPN. The changes of soil temperature in the 0-10 cm layers showed similar seasonal trends, with gradually increased after the seedling stage and slowly decreased from the silking stage to maturity. Similary, the SWS increased during the early growth stages and peaked at its highest level in the tasseling stage. The ridge and furrow rain-harvesting planting system treatments significantly increased grain yield and above-ground biomass compared with the conventional flat planting treatments, and the order of importance for increasing grain yield and above-ground through nutrient management was integrated nitrogen fertilizer and crop straw > nitrogen fertilizer. Regardless of 2020 and 2021, the grain and straw N content, plant N uptake, WP, NUPE, AEN, and PFPN also similar with the trend of grain yield and above-ground, which were highest in RFSN, followed by CPSN, FPN and CPN, FP and lowest in CP. However, the effect of fertilizer was more obvious under RFSN compared with CP, thereby indicating a positive interaction between water and integrated nutrient management. Nevertheless, the contents of the four soil organic carbon and nitrogen fractions (DOC, EOC, MBC and MBN) were determined in the 0-20 cm soil layer with similar trends: RFSN>CPSN>RFN>CPN>RF>CP. Eventually, the results of gray relation analysis and entropy weighted TOPSIS model demonstrated that ridge and furrow rain-harvesting planting system as a suitable pattern for optimizing water use with application of maize straw 1.5 t ha^-1^ and application of chemical nitrogen fertilizer of 180 kg ha^-1^ (RFSN) was as a promise strategy in enhancing yield and utilization efficiency on the Loess Plateau in China based on the comprehensive consideration of soil temperature, soil water storage, yield productivity, uptake, water-N productivity and soil carbon fraction, which is attractive because it is simple to implement, enhances soil fertility, and improves overall nutrient utilization.

## Data Availability

Datasets used in the current study are available from the correspondingauthor on reasonable request.

## Abbreviations

CP: Conventional flat planting
RF: Ridge and furrow rain-harvesting planting system
SWS: Soil water storage
NUPE: Nitrogen uptake efficiency
AEN: Agronomy efficiency of nitrogen
PFPN: Partial factor productivity of nitrogen fertilizer
DOC: Dissolved organic carbon
EOC: Easily oxidizable organic carbon
MBC: Microbial biomass carbon
MBN: Microbial biomass nitrogen
